# Celeberrimo Doctori G. Pearson, &c. S. P. D.

**Published:** 1800-08

**Authors:** J. C. W. Juncker

**Affiliations:** Professor Medicinae in Academia Regis Borussorum Halensi Ordinarius, &c. Halae Magdeburgicae


Celeberrimo Doctori G. PEARSON, &c.

S. P. D.

Med. et Chir. Dr. J. C. W. JUNCKER,

Profeſſor Medicinae in Academia Regis Boruſſorum Halenſi Ordinarius, &c. Halae Magdeburgicae, d. 17 Junii, 1800.

COGNITUM forte habebis, vir celeberrime, ſocietatem medicorum Germaniæ ac Helvetiae mecum conveniſſe, ut miſeriis humanis, quae a veneno varioloſo proveniunt, limites, quoſcunque ponere liceat, ponantur. En! brevem hujus inſtituti hiſtoriam.

Anno 1791, in urbe noſtra morbi graſſabantur maligni, ſic dicti nervoſi. Inſitio variolarum hoc tempore temere inſtituta (neglectis nempe regulis, quae ad evitandam mali propagationem ſpectant) anſam praebebat epidemiae variolarum. Haec invaſit homines 2151; ex his necavit 430; alios 7 videndi facultate privavit, et multifaria denique mala reliquit in hominibus 280. Dicto anno et partim etiam anno ſequente ſingula urbis noſtrae aedificia quin et pagos proxime vicinos exploravi reſpectu variolarum; auditorum nempe 20 auxilio adjutus. Quot et quantas miſerias malum varioloſum ejuſve inſitio temere inſtituta induxerit, hoc modo (dicta nempe exploratione aedificiorum, &c.) ſatis exacte poterat cognoſci. Mala, quae tum vidi, animum moverunt mirifice. Jam fretus multorum (potiſſimum medicorum) promiſſis, &c. fore ſperabam, ut noſtrae urbis incolae optimis conſiliis, quibus malum varioloſum vel mitigari vel arceri poſſet, aures praebituri ſint benevolas. Egregio exemplo noſtrates præire alioſque vicinos ad imitationem utiliſſimam, tali exemplo praegreſſo, eo facilius movere poſſe putabam. Edidi in hunc finem primis menſibus anni 1792, librum de variolis, in uſum praeprimis Halenſium conſcriptum.* At ſpes me fefellit. Paulo poſt, cum hiſtoriam morbi cujuſdam (Doctoris nempe Bahrdtii) deſcriberem, † querendum jam erat de auxilio medicorum quorumdam circa variolas denegato, &c. (Cfr. libell. infra laudatus, No. 2, p. 54.)

Seriori inſtituta hac de re meditatione, aliam viam incedendam et ad omnes reliquos, quamvis remotiſſimos, *humanos* tamen medicos, nec non ad magnates quoſcunque, &c. ſenſim confugiendum eſſe, ſentiv. In libello itaque anno 1795 edito,‡ medicorum Germaniae attentionem dirigere ſtudui ad majus quoddam

* *J. C. W. Juncker's*, &c. Gemeinnützige Vorſchläge und Nachrichten über das beſte Verhalten der Menſchen in Rückſicht der Pockenkrankheit. *Erſter Verſuch*. Halle 1792. Gr. 8. ſ. 236 und 96. (Jetzt in Commiſſion Halle bey Hemmerde. Pr. 1 thaler.)

† Etwas über die Weinbergſkrankheit des verſtorbenen Doctor Bahrdts und ähnlicher noch lebender Kranken. Den Nichtärzten zur freundſchaftlichen Warnung mitgetheilt von Dr. *J. C. W. Juncker*, &c. Halle in Commiſſion bey Hemmerde, 1792. Kl. 8. ſ. 55. Pr. 5 groſchen.

‡ Dr. *J. C. W. Juncker's*, &c. Gemeinnützige Vorſchläge und Nachrichten über die Pockenkrankheit. Für Deutrchlands Aerzte. Ein Vorſchläge aus der Volokſarzneywiſſenſchaft. Halle bey Hemmerde, 1795. Gr. 8. ſ. 67. Pr. 5 groſchen. (Item ſub titulo: Gemeinnützige Vorſchläge. *Zweyter Verſuch.*)

quoddam de variolis opus proxime proditurum, in quo omnia, quae huc pertinent, uberius essent illustranda.

Id quod prodiit sub titulo inferius laudato,* anno 1796. In hoc opere viam designasse mihi videor, qua quidem medicis illam incedentibus certissime datum foret, successivam praeparare et ad finem perducere variolarum exstirpationem; et 84 objectiones, huic exstirpationi oppositas, refutavi, ad viam nempe dictam respiciens. Simul autem ex omnibus Germaniae medicis petii, ut, quidquid circa hanc viam monendum esse ipsis videretur, mecum communicarent.

His precibus jam nunc multo plures satisfecerunt medici, quam 300; inter quos plures eminent nostri instituti egregie participes.—Plures simul nummos dono dederunt, unde enatum est, quod optaveram, *aerarium contra variolas generale* seu *commune* (h. e. variolarum remotioni in universum dicatum. Neque enim *singulis* quibusdam, sed *omnibus* locis illud aerarium dicavimus, illudque opponimus aerariis *localibus*, quibus fore speramus, ut in hoc illove *loco* incolae ejusdem aliquando sibi prospiciant. Dictum vero *aerarium commune* jam nunc circiter continet duo millia imperialium, numeratis nempe simul nummis subscriptionis lege promissis.)

Ut vero judicia medicorum facilius transmitti potuerint, ex omnibus fere, qui Germaniam regunt, magnatibus precatus sum, ut, finito quovis anno, numerum eorum, qui praeterito anno in suis regionibus mortui essent variolis, aliaque, quae variolarum respectu memoratu digna viderentur medicis seu verbi divini ministris, colligenda et haec denique omnia collecta sive summatim excerpta mihi transmittenda curarent. Responderunt his votis plurimi iique potentissimi Germaniae principes; Imperator v. g. Germaniae; Rex Borussorum; Elector Saxoniae; Duces Saxoniae; Magnates Harsiae; Megapolitani; Auhaltini, aliique multi.

Tot itaque adminiculis instructus inde ab anno 1796 *periodicum opus* edidi, ut quovis anno vel semestri innotesceret, quales in hac causa progressus fecerit societas medicorum. Cujus quidem operis huc usque prodierunt volumina septem.† Volumen

---

* Dr. *J. C. W. Juncker's*, &c. Gemeinnützige Vorschläge wider die Pockenkrankheit. *Dritter Versuch.* Für möglichst alle Aertze, die der deutschen sprache kundig sind; zur sammlung ihrer Gutachten hierüber. Halle bey Hemmerde. Gr. 8. s. 502. Pr. 1 thaler 8 groschen.

† Archiv der Aerzte und Seelsorger wider die Pockennoth. *Erstes Stück.* Michaelmesse 1796. Heraus gegeben von Dr. *J. C. W. Juncker*, &c. Leipzig in der Weygandschen Buchhandlung, 1796. Gr. 8. s. 296. Pr. 18 groschen. *Zweytes Stück.* Ostermesse 1797. Gr. 8. s. 306. Pr. 22 groschen. *Drittes Stück.* Michaelmesse 1797. Gr. 8. s. 218. Pr. 18 groschen.

Volumen octavum prodibit tempore nundinarum Lipſienſium, quae ſequuntur. Novo, quod proximum eſt, ſeculo idem opus periodicum, titulo paululum mutato, continuabitur. (*Neues* Archiv wider das Pockenelend, &c.)

Jam vero comparatis Germaniae medicorum ſuffragiis, meum eſſe putavi, in unum colligere, quae omnibus iſtis medicorum conſiliis conſentanea ſint, quaeque *noſtris* temporibus et adeo diverſis locis Germaniae, &c. reſpondeant. *Et haec quidem omnia* (miſſis ceteris, in quibus medici diſſentiunt, &c.) *cum omnibus Germaniae magnatibus praeſenti anno* ſeu certe primis futuri anni menſibus communicabo.

Reſponſiones magnatum exſpectamus ſequente anno 1801. Quibus collectis novum de ſucceſſiva variolarum exſtirpatione opuſculum lingua Latina aeque ac Francogallica conſcriptum, *omnibus* itidem *exteris*, quibuſcunque licebit, *magnatibus* tranſmittendum curabit ſocietas medicorum. Brevem hujus libelli mentionem jam feci in *Mémoire adreſsé au Congrès de Raſtadt, concernant la petite vérole* menſe Maii 1798. De his autem et ſimilibus ſuo tempore pluribus te reddam certiorem.

Utinam et tibi placeret, vir praeſtantiſſime, *finito quovis anno* mecum communicare, quae tibi nuperius digna viſa ſint memoratu circa variolas *in patria tua!* (quinam v. g. libri huc pertinentes anno quocunque praeterito prodierint? quae *remedia* horrendo huic veneficio oppoſita ſint *vi legum* vel *ſuaſu medicorum? &c.*

Et hoc quidem *primum* eſt, quod ex te precor. *Delectes me hujus generis epiſtolis finito quovis anno!*

*Alterum* vero momentum, cujus cauſa has litteras conſcripſi, *inſitionem* ſpectat *variolarum vaccinarum.* Legi de his ſequentes libros: 1. An Inquiry into the Cauſes and Effects of the Variolæ Vaccinæ, &c. by *E. Jenner*, Lond. 1798. (vers. in linguam Germanicam, *Ballhorn*, Hannover, 1799; in linguam Latinam *Careno*, Vindob. 1799;) 2. Further Obſervations, &c. by *E. Jenner*, Lond. 1799. 3. An Enquiry concerning the Hiſtory of the Cow-pox, &c. by *G. Pearſon.* London, 1798; (vers. in linguam Germanicam, *Küttlinger.* Nürnberg, 1800;) 4. Reports of a Series of Inoculations for the Variolæ Vaccinæ, or Cow-pox, &c. by *W. Woodville.* Lond. 1799. (Vers. in linguam Germanicam, *Frieſe.* Breſlau, 1800.)

Jam

---

*Viertes Stück.* Neujahrſmeſſe 1798. Gr. 8. ſ. 291. Pr. 1 thaler 4 groſchen. *Fünftes Stück.* Oſtermeſſe 1798. Gr. 8. ſ. 320. Pr. 22 groſchen. *Sechstes Stück.* Michaelmeſſe 1798. Gr. 8. ſ. 304. Pr. 1 thaler. *Siebentes Stück,* Michaelmeſſe 1799. Gr. 8. ſ. 352. Pr. 1 thaler 4 groſchen.

Jam vero in votis eſt, ut infantibus quibuſdam Halenſibus, quos in deliciis habeo, aeſtate hujus anni ſive ejuſdem autumno inſerere queam variolas vaccinas. Ne vero decipiar et materiem forte accipiam a variolis vaccinis *ſpuriis* depromptam aliove reſpectu ſive perniciofam ſive non ſatis efficacem: *Ardentiſſime ex te precor*, Ut mihi, quam proxime fieri id queat, tranſmittendam cures materiem his votis apprime conſentaneam. Quem igitur in finem ſollicitus, quaeſo, ſis de homine ceterum *ſaniſſimo*, *qui poſt inſitionem materiei*, e vacca puſtulis vaccinis laborante *recenter* depromptae, variolas vaccinas *veras leniter* (ſueta, volo, non anomola ratione) perpeſſus ſit. Materiem ex HOC HOMINE depromptam mihi, precor, tranſmittendam cures, vaſe *v. g.* vitreo bene obturato et capſula inſuper *v. g.* lignea munito incluſam. In ſchedula annexa tua manu benevole teſteris, materiem hoc vaſe incluſam *vere talem eſſe qualem optaverim*, eamque igitur ita eſſe comparatam, ut ab inſitione ejuſdem (ceterum rite inſtituta) ſoliti et ſalutares eventus expectari queant.

Quibuscunque impenſis hujus generis labor anſam forte praebiturus ſit, gratiſſimo animo compenſabo. — Si forſan *cautelae quaedam*, quae *inſitionem hanc* ſpectant, ad notitiam tuam *nuperius* veniſſent, quarum in libris ſupra laudatis mentio facta *non* eſt: *has quoque cautelas* mecum benevole communices!

Vale. Fauſta quaevis Tibi apprecor!

P. S.

*Adreſſe.*

Litteris Tuis extus quaeſo inſcribas:

Dem Herrn,

Dr. *J. C. W. Juncker*,

Profeſſor der Medicin auf der Koeniglichen Preuſſiſchen Univerſität.

Nebſt einem Päckchen, bezeichnet:

*Medicin* aus London, an H. P. J. zu Halle.*

Zu HALLE, (bey *Leipzig.*)

* Haec verba inferius et ſiniſtrorſum poſita ſignificant: litteras tuas comitari vaſculum, quod contineat medicinam Londinenſem, et quod ſignatum ſit verbis: an H. P. J. zu Halle.

---

The foregoing Letter was ſent to Dr. PEARSON, and by him given to the Vaccine Pock Inſtitution, from which we have permiſſion to inſert it. — EDIT.

